# Lanthanide-Doped Upconversion-Linked Immunosorbent Assay for the Sensitive Detection of Carbohydrate Antigen 19-9

**DOI:** 10.3389/fchem.2020.592445

**Published:** 2021-02-26

**Authors:** Chaohui Zhou, Zhongyun Chu, Wenyue Hou, Xiuying Wang

**Affiliations:** ^1^The Education Ministry Key Laboratory of Resource Chemistry, Shanghai Key Laboratory of Rare Earth Functional Materials, School of Chemistry and Materials Science, Shanghai Normal University, Shanghai, China; ^2^School of Intellectual Property, Xihua University, Chengdu, China

**Keywords:** UCNPs, immunosorbent assay, cancer biomarker, CA19-9, immunodetection

## Abstract

Lanthanide-doped upconversion nanoparticles (UCNPs) have attracted considerable attention in detection of biological analytes and bioimaging owing to their superior optical properties, including high photochemical stability, sharp emission bandwidth, large anti-Stokes shifts, and low toxicity. In this work, we fabricated UCNP-linked immunosorbent assay (ULISA) for the sensitive detection of carbohydrate antigen 19-9 (CA19-9). The design is based on amino-functionalized SiO_2_-coated Gd-doped NaYF_4_:Yb^3+^,Er^3+^ upconversion nanoparticles (UCNPs@SiO_2_-NH_2_) as a direct background-free luminescent reporter; a secondary anti-IgG antibody (Ab_2_) was conjugated to the surface of UCNPs@SiO_2_-NH_2_ (UCNP-Ab_2_), and UCNP-Ab_2_ was used for specific targeting of CA19-9. The UCNPs were well characterized by TEM, SEM, XRD, FT-IR, and UV-vis. The detection process was similar to enzyme-linked immunosorbent assay (ELISA). UCNPs were used as signal transducer to replace the color compounds for an enzyme-mediated signal amplification step. An anti-CA19-9 primary antibody (Ab_1_) was fixed for capturing the CA19-9, and the fluorescence signal was obtained from the specific immunoreaction between UCNP-Ab_2_ and CA19-9. Under optimum conditions, this ULISA shows sensitive detection of CA19-9 with a dynamic range of 5–2,000 U/ml. The ULISA system shows higher detection sensitivity and wider detection range compared with the traditional ELISA for CA19-9 detection. This strategy using UCNPs as signal transducer may pave a new avenue for the exploration of rare doped UCNPs in ELISA assay for clinical applications in the future.

## Introduction

Cancer has been the leading cause of global death. Among various types of cancers, pancreatic cancer with extremely high mortality and poor prognosis is ranked the 4th leading cause of cancer death in the USA (Wolfgang et al., 2013; Siegel et al., 2019). In China, the incidence and mortality of pancreatic cancer are also increasing (Chen et al., 2016). The late diagnosis of cancers was deemed to be the leading cause of high mortality rate because of the lack of early symptoms and appropriate tools to detect pancreatic cancer at an early stage. In order to decrease mortality and improve survival of pancreatic cancer patient, it is crucial to confirm the lesion at an early surgically resectable stage (Qian et al., 2019). The CA19-9 has been widely used as a clinical common tumor-specific biomarker for diagnosing pancreatic cancer (Xu et al., 2019). CA19-9 is also the only approved biomarker to guide and direct therapy, prognosis, and diagnostics by the US Food and Drug Administration (FDA) (He et al., 2015). Elevated levels (>37 U/ml) of CA19-9 are thought to be closely related to pancreatic cancer (Wu et al., 2013). Thus, the accurate monitoring of CA19-9 has been a vital tool for early diagnosis of pancreatic cancers (Kaur et al., 2012; Singhi et al., 2019).

Enzyme-linked immunosorbent assay (ELISA) is an extensively used immunological assay in medical diagnostic for measuring the proteins, peptides, antibodies, haptens, hormones, illegal drugs, cells, and their metabolites in biological samples. ELISA presented a simple and reliable analytical tool for the detection of target biological analytes (Kwong et al., 2013). However, there are some limitations that hamper its routine application in the clinical detection of cancer biomarkers due to the ELISA tests being mainly based on the spectroscopic detection of a chromogenic substrate that yields a measurable color product during the assay. This colorimetric signal readout significantly affects the accurate detection of the target biomolecules as the color intensity is proportional to the concentration of target analytes. Unfortunately, the sensitivity of colorimetric readout is relatively lower compared with other signal readout such as fluorescence, which will decrease the sensitivity of the traditional colorimetric ELISA assay. Additionally, some color compounds in the biological samples will also interfere with the color output (Acharya et al., 2013; Tong et al., 2013; Xianyu et al., 2014; Gao et al., 2020).

Recently, nanoparticles (NPs) as signal amplifiers have been widely used as strategies to improve the performance of the traditional colorimetric output-based ELISA. Quantum dots, fluorescent dye-doped polymer or silica NPs, catalytic NPs, metal NPs, and magnetic NPs have been successfully used in ELISA, remarkably improving the sensitivity of ELISA (Osterfeld et al., 2008; Jans and Huo, 2012; Tong et al., 2013). Rare-earth (RE)-doped upconversion luminescent nanoparticles (UCNPs) as an optical background-free luminogens have exhibited enormous potential in immunodetection owing to its possession of large anti-Stokes shifts, high photochemical stability, long photoluminescence (PL) lifetimes, and low toxicity (Wang and Tanner, 2010; Liu et al., 2013; Zhao et al., 2013). UCNP-based linked immunosorbent assays have been designed for highly sensitive detection of clinical biomarker such as cardiac troponin I (Sirkka et al., 2016), multiple mRNAs (Hu et al., 2017), and prostate-specific antigen (PSA) (Farka et al., 2017). However, UCNP-based linked ELISA system for CA19-9 detection has not been developed.

**Scheme 1:**
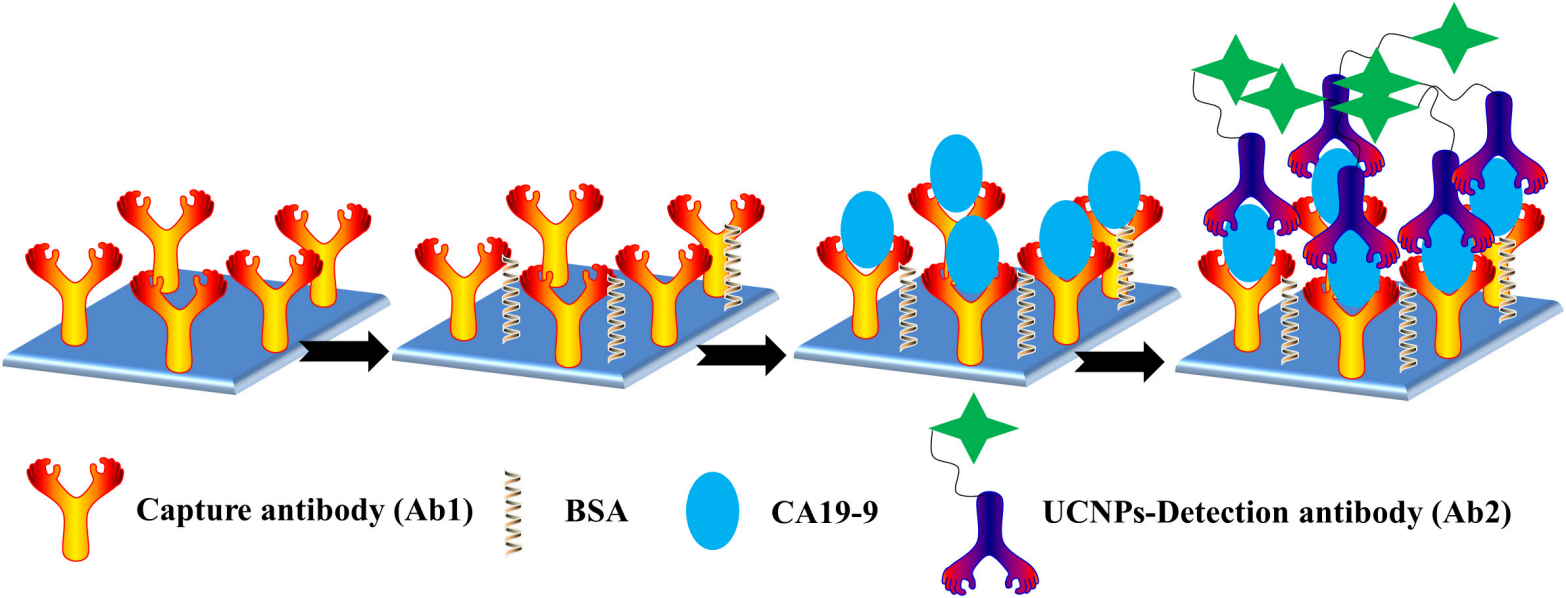
Schematic representation of the ULISA for CA19-9 detection in 96-well polystyrene (PS) plates.

In this work, we developed a new UCNP-based linked immunosorbent assay (ULISA) for the sensitive detection of CA19-9. The system was constructed based on amino-functionalized SiO_2_-coated Gd-doped NaYF_4_:Yb,Er UCNPs (UCNPs@SiO_2_) that works as signal transducer to provide the fluorescence signal. UCNPs were coated with silica by inverse emulsion method and followed by functionalization with amino groups on the surface (Hlaváček et al., 2014). Anti-mouse IgG antibodies were conjugated to the surface of UCNPs@SiO_2_-NH_2_. The strategy for detection of CA19-9 is illustrated in Scheme 1. After optimizing testing condition, this ULISA could achieve sensitive and rapid detection of CA19-9. This design strategy of using UCNPs as the signal transducer to provide fluorescence as an alternative output to color intensity will be extended to wide-range applications in the area of immunodetection and disease diagnosis.

## Experimental

### Materials and Characterization

All the applied chemicals such as Y_2_O_3_, Yb_2_O_3_, Er_2_O_3_, and Gd_2_O_3_ in this work maintained high purity (purity ≥98%) were provided by Sigma-Aldrich (USA) and without further purification. Ammonium fluoride (NH_4_F), absolute tetraethyl orthosilicate (TEOS), and oleic acid (OA) were obtained from Shanghai Reagent Company. The washing buffer (pH = 9.6) employed in immunosorbent assay was doped with solution components such as 50 mM NaH_2_PO_4_/Na_2_HPO_4_, 0.01% Tween 20, and 0.05% NaN_3_. All aqueous solutions in experiments were prepared in distilled water (DIW, Millipore-Q, 18.2 MΩ).

The measurements of zeta potential were performed using Malvern Zetasizer Nano ZS90; the morphology and size of nanoparticles were characterized on a transmission electron microscopy (TEM, JEOL JEM-2100) and field emission scanning electron microscope (FESEM, LEO1530). Meanwhile, UV-visible (UV-vis) absorption spectra were recorded on a Varian Cary-Eclipse 500 apparatus. Nicolet 380 spectrometer system was applied to record Fourier transform infrared (FT-IR) spectra.

### Preparation of UCNPs-SiO_2_-NH_2_

The nanostructured UCNPs-SiO_2_-NH_2_ was fabricated in the following procedures:

1. Synthesis of lanthanide-doped UCNPs (Hou et al., 2015). In the first step, a volume of 20 ml of ethanol and 30 ml of OA solution were added to the beaker. Subsequently, 5 ml of DIW and NaOH (1.2 g) were added under a steady magnetic stirring rate to obtain a transparent color. Immediately, 3.2 ml of NH_4_F (2 mol/L) solution was quickly loaded into the above system. After stirring for 10 min, a certain amount of RECl_3_ solution (0.4 M, 4 ml) was gradually dropped. It should be noted that RE referred to the mixture of the above rare earth elements, and the ratio of the amount of the substance was Y:Gd:Yb:Er = 9:30:10:1. After stirring for 30 min, the dispersion was completely transferred to a hydrothermal reaction vessel with hydrothermal treatment at 180°C going on for 2 h, and the upper layers and the lower sample nanoparticles were carefully separated and dissolved in cyclohexane, followed by aspiration with absolute ethanol before centrifugation. All of the nanomaterials were rinsed repeatedly with water and ethanol for several cycles to remove unreacted precursor and then subjected to a freeze-drying process overnight.
2. The silica coating on UCNPS (UCNPs@SiO_2_). The reverse microemulsion method was used to achieve the purpose of encapsulating UCNPs (Hlaváček et al., 2014). The specific route was to mix 2-(2-[4-(1,1,3,3-tetramethylbutyl) phenoxy] ethoxy) ethanol (Triton X-100), *n*-hexyl alcohol, and cyclohexane in a ratio of 4:1:1 and adding a certain amount of DIW to form a transparent and stable microemulsion system. Then, nanoparticles were thrown into the system, dispersed, and pipetted into the flask for vigorous magnetic stirring. Afterwards, a portion of concentrated ammonia water was taken and added dropwise; when it was uniformly dispersed, TEOS was continuously placed into the system under continuous stirring for 10 h. Standing aging and subsequent ethanol washing and preservation steps were expected.
3. Surface amino functionalization of UCNPs (UCNPs@SiO_2_-NH_2_). A mixture of methanol and glycerol in a volume ratio of 5:3 was used to disperse the UCNPs@SiO_2_ with magnetic stirred at 65°C. Afterwards, 10 ml of aminopropyltriethoxysilane (APTES, Sigma-Aldrich) was added for 5 h of continuous reaction; the nanocomposites were washed with ethanol and phosphate buffer solution (PBS, pH = 7.4) for three times.

### Conjugation of UCNPs@SiO_2_-NH_2_ and Secondary Antibody

The UCNPs-SiO_2_-NH_2_ were conjugated to a secondary antibody *via* standard EDC/sulfo-chemistry (Ko and Lim, 2014). In a typical synthesis, the presynthesized UCNPs@SiO_2_-NH_2_ were dispersed in 10 ml of PBS solution at room temperature under slight magnetic stirring for 2 h. Then, 1-ethyl-3-(dimethylaminopropyl) carbodiimide (EDC) and *N*-hydroxysuccinimide (NHS) mixture (1:1) were added and mixed for 1 h. The secondary antibody (Ab_2_, Abcam) was introduced into the system and kept going for another 2 h under regular shaking. The prepared sample UCNPs@SiO_2_-Ab_2_ was finely purified by multiple centrifugation to remove unreacted solvents or impurities, then the complex was dispersed in PBS for next experiments.

### UCNP-Based Linked Immunosorbent Assay

First, the optimal coating concentration of monoclonal antibodies was determined using a transparent and clean 96-well plate with pretty protein binding capacity (Corning, Germany). Based on the concentration test, each well in plate was coated with primary antibodies (Ab_1_, Abcam) at an optimal concentration of 2 μg/ml for overnight (4°C), except for this, all subsequent procedures were launched under room temperature. Subsequently, the plate was rinsed with 250 μl of washing buffer four times manually. Naturally, the remaining free sites on the plate were blocked with 1% BSA solution (PBS, pH = 7.4) for 2 h. After washing procedures, the standard CA19-9 solution (100 μl) with various concentrations was added to each well and incubated for 2 h. Similarly, the unbound Ab_1_ was rinsed off with PBS buffer. Immediately, UCNPs@SiO_2_-Ab_2_ was introduced into each well and further incubated for 2 h and following by washing. Finally, the upconversion luminescence signals were recorded under a custom-built upconversion microplate reader equipped with a 980-nm laser transmitter.

## Results and Discussion

### Characterization and Surface Modification of UCNPs

The application of ULISA for detection strongly relies on the performance of the luminescent reporter. Among the various developed upconversion materials, NaYF_4_:Yb/Er nanoparticles with hexagonal crystal structure express greater upconversion efficiency than its cubic crystal form. Moreover, the dispersibility and stability of nanomaterials are also key factors in the assay. In our study, the as-prepared UCNPs with oleic acid as a stabilizer were coated with silica shell, which not only enhance the dispersion of UCNPs but also provide a large number of sites for subsequent functional modification.

**Figure 1:**
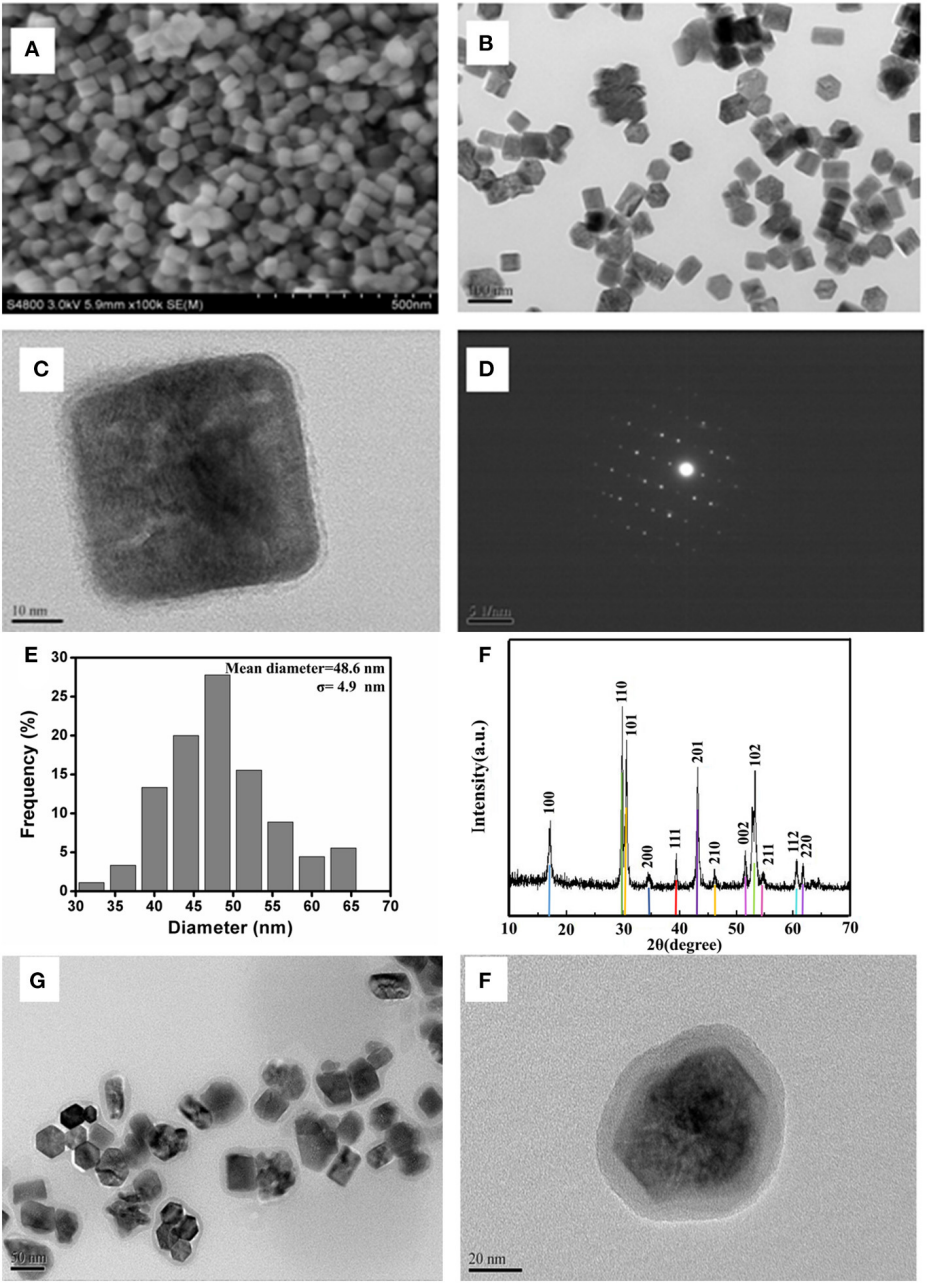
**(A,B)** SEM and **(C)** TEM images of UCNPs. **(D)** Selected area electron diffraction pattern of UCNPs, **(E)** the size distribution of UCNPs, **(F)** XRD patterns for UCNPs (up line) and standard powder (bottom bar) JCPDS card, and **(G, H)** the TEM image of the silica-coated UCNPs with different resolution ratios.

As shown in Figure 1, the hexagonal phase structure of UCNPs was clearly shown in the FESEM image (Figures 1A,B). Furthermore, both the TEM image (Figure 1C) and the FESEM images verified the good dispersibility of the nanoparticles. Additionally, it has uniform size distribution with a homogeneous diameter of 48.6 ± 4.9 nm (Figure 1E). Notably, UCNPs also emerge as a considerable degree of crystallinity and sharpness. The selected-area electron diffraction pattern (Figure 1D) exhibited a single crystal diffraction ring, which is in accordance with the X-ray diffraction results. The corresponding conspicuous peak diffraction (Figure 1F) such as the angles of 17.08°, 29.85°, 30.42°, 43.24°, and 53.75° corresponds to the planes of (100), (110), (101), (201), and (102), respectively, suggesting the pure hexagonal phase of UCNPs. For upconversion materials, the luminescence properties of UCNPs in ULISA applications must be taken into consideration. Under the irradiation with laser at 980 nm, the upconversion luminescence spectrum is recorded in Supplementary Figure 1. The three emission peaks at about 520, 540, and 660 nm were observed, and the most intensive fluorescence is centered at 540 nm, which could be ascribed to the typical green region. The full UV spectrum of UCNPs is shown in Supplementary Figure 2. In order to improve water dispersibility and stability of UCNPs, the one-step silica-coating protocol was conducted using the reverse microemulsion method. In the previous exploration process, the one-step silicon coating easily lead to agglomeration of UCNPs, a two-step protocol was used to prepare a more stable and compact silica shell. Figures 1G,H shows the TEM image of the silica-coated UCNPs. It was clear that each nanoparticle was coated with about 5 nm compact silica shell.

**Figure 2:**
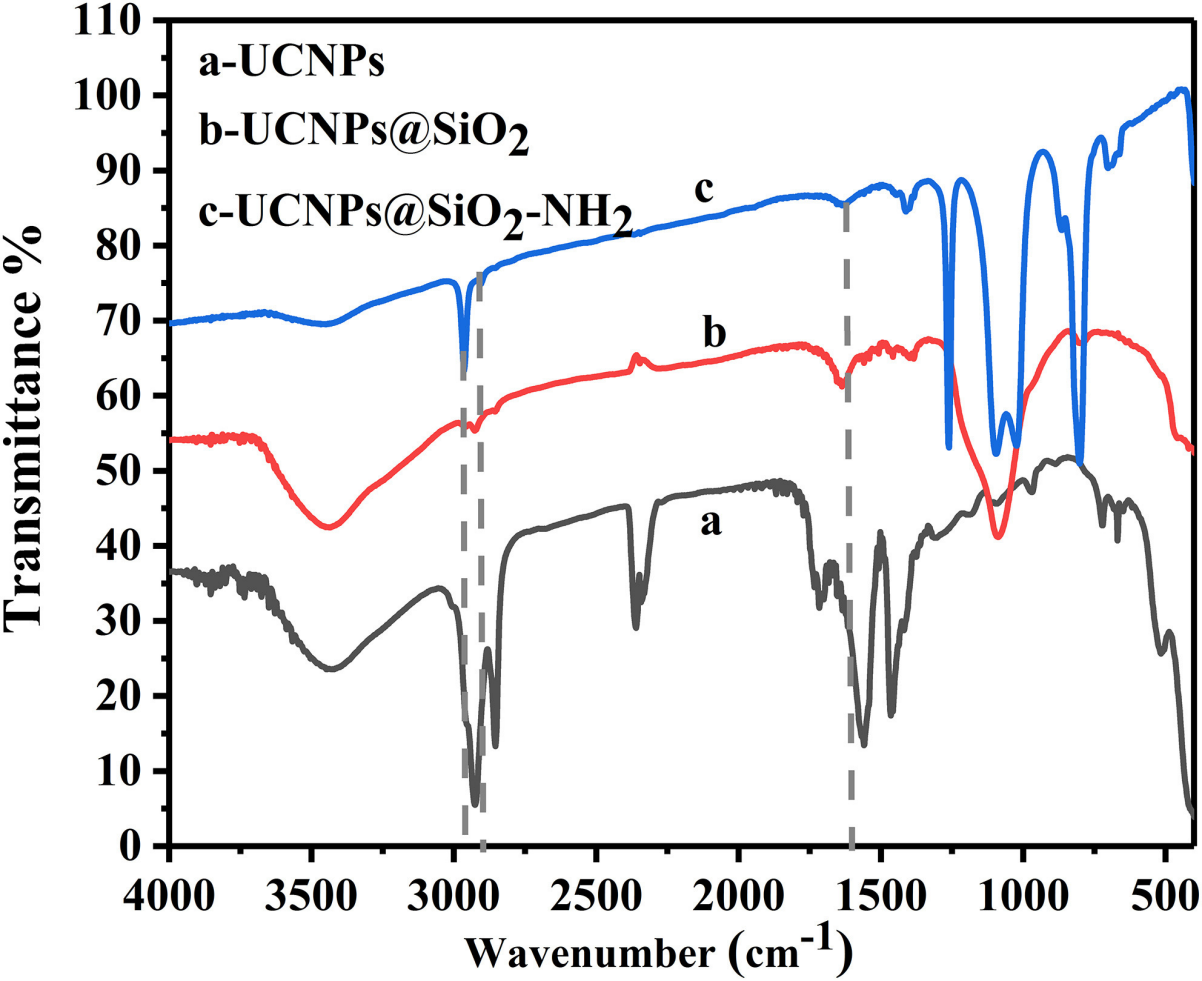
The FT-IR spectra of UCNPs before and after SiO_2_ and NH_2_ modification.

In order to facilitate the coupling of UCNPs to Ab_2_, the amino functional modification of the surface was conducted. In the first step, the potential trend (Supplementary Figure 3) in different modification stages was measured. Owing to hydroxyl groups on the surface of the silica coating, it displayed a potential change from positive (+36.2 mV) to negative (−31.7 mV). However, after modification with APTES, the potential changed to +33.2 mV, which could account for the amino group of the surface of the silica. Thus, this result demonstrated that amino was successfully conjugated to the surface of UCNPs@SiO_2_. Additional characterization was further conducted to verify the successful modification. By means of FT-IR spectroscopy (Figure 2), the bands at 1,094 and 3,420 cm^−1^ are assigned to stretching vibrations of –Si–O–Si and O–H in UCNPs@SiO_2_; this fully confirmed the successful wrapping of silica. In the nanocomplex UCNPs@SiO_2_-NH_2_, the characteristic absorption bands of N–H (1,641 cm^−1^) bending vibrations and C–H (2,974 cm^−1^) stretching vibrations indicate the existence of amino group (Huang et al., 2010). The successful coating of SiO_2_-NH_2_ onto the surface of UCNPs not only enables the dispersibility of UCNPs in biological fluids but also provides a lot of attachment sites for subsequent protein conjugation steps.

### Conjugation of UCNPs@SiO_2_-NH_2_ and Ab_2_

**Figure 3:**
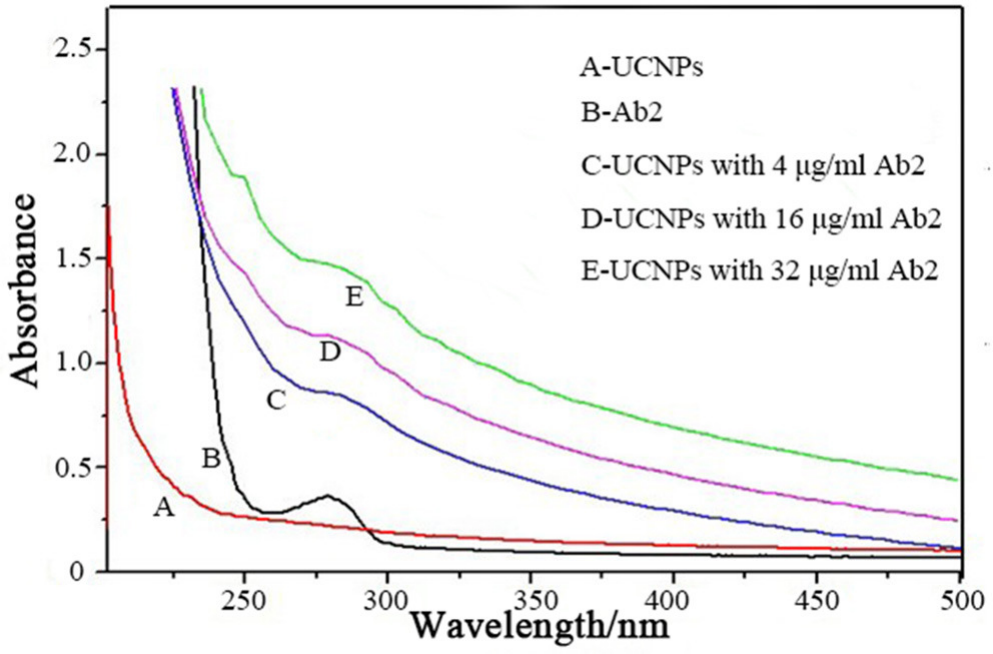
UV-vis spectroscopic characterizations of the conjugation of UCNPs@SiO_2_-NH_2_ and various concentrations of Ab_2_.

The Ab_2_ were conjugated to the surface of UCNPs@SiO_2_-NH_2_ through a standard EDC/NHS strategy. The UV-vis spectroscopy was used to characterize the conjugation of UCNPs@SiO_2_-NH_2_ and Ab_2_ (Figure 3). The characteristic absorption peak of Ab_2_ was observed at 280 nm, which is comparable with the no obvious absorption peak of the UCNPs@SiO_2_-NH_2_. After conjugation of Ab_2_ to the surface of UCNPs@SiO_2_-NH_2_, the absorption peak at 280 nm was observed and the intensity of absorption band increased with the concentration of conjugated Ab_2_. The results indicated that Ab_2_ was successfully conjugated to the surface of UCNPs@SiO_2_-NH_2_.

### Optimization of Concentration of Ab_1_

**Figure 4:**
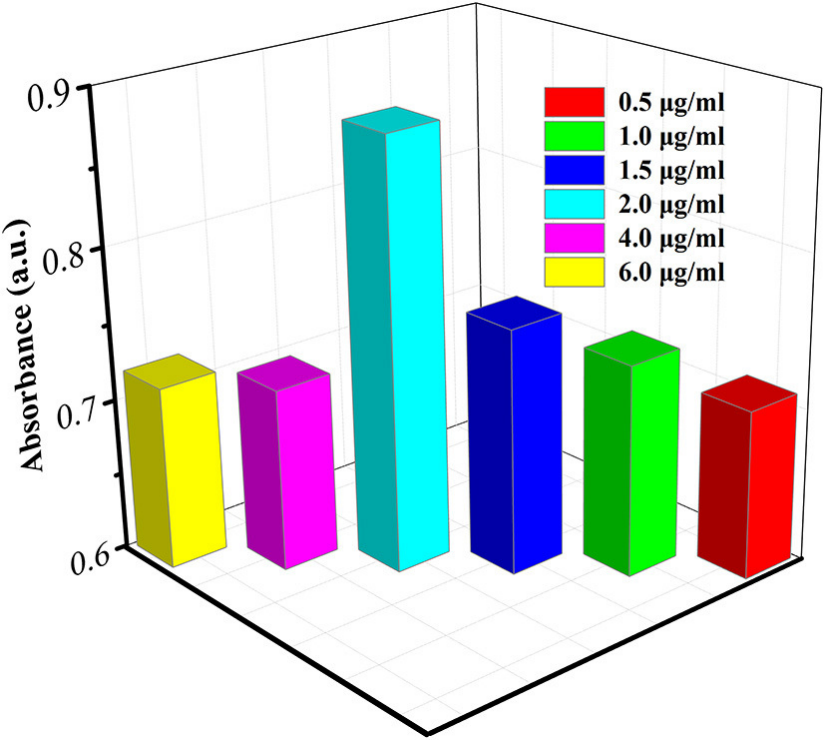
Fine tuning CA19-9 detection by using various concentrations of Ab_1_.

The coating of the antibody on the plate is based on the physical adsorption of the protein by polystyrene plastic. The amount of antibody coated on the experimental plate directly determines the detection limit of this experiment, which has a great impact on the results. Therefore, it is necessary to determine the optimal concentration of Ab_1_. In detail, firstly, the different concentrations of Ab1 (0.5, 1, 1.5, 2, 4, and 8 μg/ml) were immobilized in plate; subsequently, the excess horseradish peroxidase (HRP)-labeled Ab_2_ were added and incubated, and TMB substrate solution was added to the microwell plate after washing off the unreacted HRP-labeled Ab2. The absorbance of the reaction solution was monitored As shown in Figure 4, with the increase of the concentration of Ab_1_, the absorbance gradually increased and the absorbance value reached a maximum at the concentration of 2 μg/ml. As the concentration continued to increase, the absorbance value gradually reduced, implying that there is the largest Ab_1_ adsorption capacity when the concentration of Ab_1_ reached 2 μg/ml. Therefore, 2 μg/ml was used as the optimal concentration of Ab_1_ in the following study.

### Sensitivity of the UCNP-Based Linked Immunosorbent Assay

**Figure 5:**
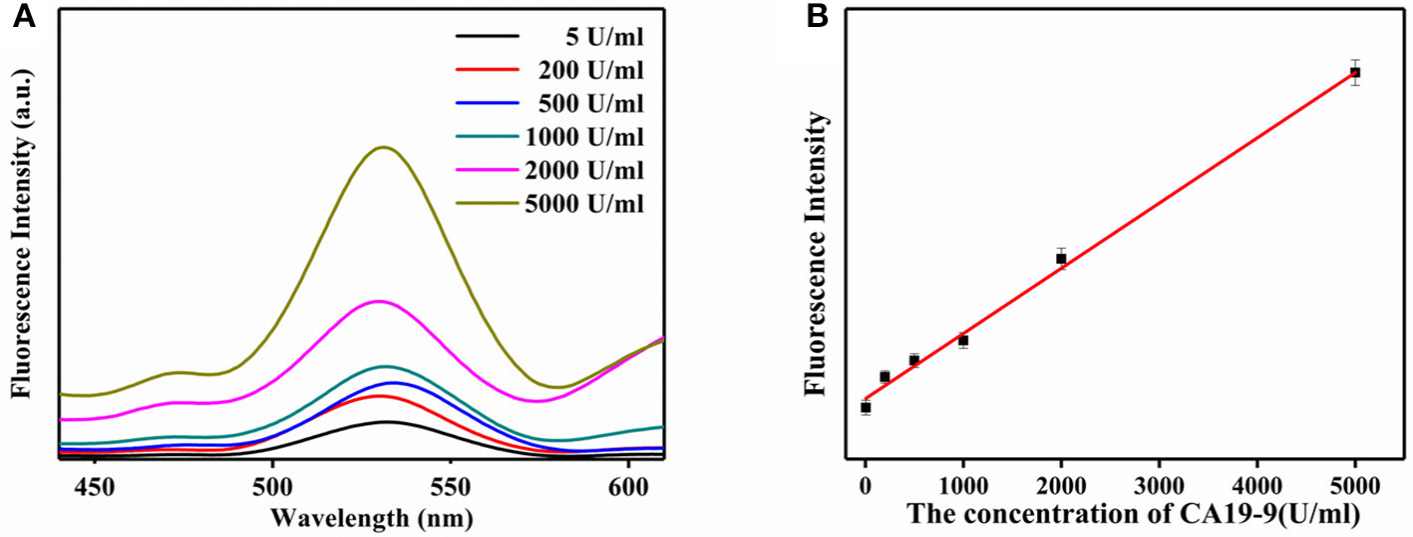
**(A)** Upconversion luminescence spectra of the ULISA at different concentrations of CA19-9. **(B)** Calibration curve of ULISA for CA19-9 detection.

The detection of CA19-9 with ULISA was conducted similarly as traditional ELISA. Only a UCNP-Ab_2_ was used as a direct luminescent reporter to replace the color compounds for enzyme-mediated color generation. Under optimum conditions, the standard concentration of CA19-9 from 5 to 2,000 U/ml were prepared *via* serial dilution in PBS, and then this ULISA system was used for the quantitative assay. The detection results for CA19-9 are shown in Figure 5A. The fluorescence intensity of UCNPs gradually increased along with the concentration of CA19-9 from 5 to 2,000 U/ml. A linear relationship between the fluorescence intensity of UCNPs at 540 nm and concentration of CA19-9 from 5 to 2,000 U/ml was obtained; the linear range for the quantitative assay is shown in Figure 5B. The correlation equation can be calculated as *y* = 0.124*x* + 115.56, and a correlation coefficient (*R*^2^) of 0.9959 was obtained. Compared with conventional ELISA assay for CA19-9 detection, this ULISA system shows higher sensitivity and wider detection range. For example, the CA19-9 ELISA kit produced by Fujirebio Diagnostics AB exhibited good sensitivity and specificity ranging from 10 to 200 U/ml (Zhen-Nan et al., 2009). Wang's group had reported an DAS-ELISA for the determination of CA19-9 and linear range was 20–300 U/ml (Wang et al., 2014). Thus, these results suggested that UCNP-based linked immunosorbent assay exhibited high selectivity and is reliable in the detection of CA19-9, which is significant in monitoring the pancreatic cancer. UCNPs working as the signal transducer will greatly improve the assay performance of ELISA and provides an optional design strategy for the ELISA. This ULISA system would be a promising tool for the detection of CA19-9 in clinical diagnostics.

## Conclusions

In summary, we have developed a new ULISA for the detection of CA19-9. The monodisperse, high-yield, small-size, and pure hexagonal Gd-doped NaYF_4_:Yb^3+^,Er^3+^ UCNPs were successfully synthesized and coated with SiO_2_, and further functionalized with NH_2_ groups for conjugation with Ab_2_. In the presence of CA19-9, the detection signal was obtained by the specific immunoreaction between UCNPs-Ab_2_. Under optimum conditions, this ULISA shows a sensitive detection of CA19-9 with a dynamic range of 5–2,000 U/ml. The ULISA system shows higher detection sensitivity and wider detection range compared with traditional ELISA for the detection of CA19-9. This strategy using UCNPs as signal transducer may pave a new avenue for the exploration of rare doped UCNPs in ELISA assay for clinical applications in the future.

## Data Availability Statement

The original contributions presented in the study are included in the article/Supplementary Material, further inquiries can be directed to the corresponding author.

## Author Contributions

XW conceived and designed the experiments and revised the manuscript. CZ contributed to data analysis and wrote the manuscript. ZC participated in discussing the results and analyzing the data. WH performed the experiments. All authors contributed to the article and approved the submitted version.

## Conflict of Interest

The authors declare that the research was conducted in the absence of any commercial or financial relationships that could be construed as a potential conflict of interest.

## Supplementary Material

Supplementary Figure 1 Luminescence spectroscopic characterization of UCNPs upon NIR excitation at 980 nm.

Supplementary Figure 2 The full UV spectrum of UCNPs.

Supplementary Figure 3 Zeta potentials of UCNPs, UCNPs@SiO_2_, and UCNPs@SiO_2_-NH_2_.
