# Supplementary figures and images for: Lanthanide-Doped Upconversion-Linked Immunosorbent Assay for the Sensitive Detection of Carbohydrate Antigen 19-9
Source: Front Chem. 2021 Feb 26;8:592445. doi: 10.3389/fchem.2020.592445 (PMC7954120; doi:10.3389/fchem.2020.592445)

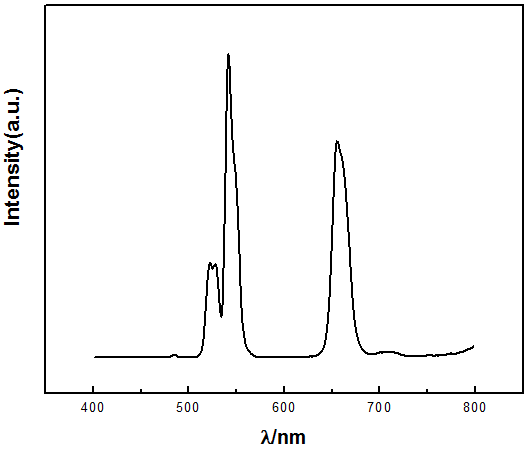

Supplement: Supplementary Figure 1 — Luminescence spectroscopic characterization of UCNPs upon NIR excitation at 980 nm. [file Image_1.tif]
